# Predictors of early and long-term mortality after ICU discharge in critically ill COVID-19 patients: A prospective cohort study

**DOI:** 10.1371/journal.pone.0293883

**Published:** 2023-11-02

**Authors:** Mariana M. S. Santos, Isabel J. Pereira, Nelson Cuboia, Joana Reis-Pardal, Diana Adrião, Teresa Cardoso, Irene Aragão, Lurdes Santos, António Sarmento, Regis G. Rosa, Cristina Granja, Cassiano Teixeira, Luís Azevedo

**Affiliations:** 1 MEDCIDS–Medicina da Comunidade, Informação e Decisão em Saúde, Department of Community Medicine, Information and Health Decision Sciences, Faculty of Medicine, University of Porto, Porto, Portugal; 2 CINTESIS@RISE–Center for Health Technology and Services Research (CINTESIS) & Health Research Network Associated Laboratory (RISE), University of Porto, Porto, Portugal; 3 Faculty of Medicine, University of Porto, Porto, Portugal; 4 Polyvalent Intensive Care Medicine Service, Centro Hospitalar de Gaia/Espinho—Vila Nova de Gaia, Vila Nova de Gaia, Portugal; 5 CriticalMed–Critical Care & Emergency Medicine, CINTESIS—Center for Health, Porto, Portugal; 6 Intensive Care Unit (UCIP), Hospital de Santo António, Oporto Hospital Center, University of Oporto, Largo Prof. Abel Salazar, Porto, Portugal; 7 CHUSJ-Centro Hospitalar Universitário S. João, Porto, Portugal; 8 Infectious Diseases Intensive Care Unit-(ID-ICU)- Centro Hospitalar Universitário S. João, Porto, Portugal; 9 Intensive Care Department, Centro Hospitalar Universitário de São João—Porto, Porto, Portugal; 10 Hospital Moinhos de Vento, Porto Alegre, RS, Brazil; 11 Research Unit, INOVA Medical, Porto Alegre, RS, Brazil; 12 Brazilian Research in Intensive Care Network (BRICNet), São Paulo, SP, Brazil; 13 Anaesthesiology Department, Centro Hospitalar Universitário São João, Porto, Portugal; 14 Department of Surgery and Physiology, Faculdade de Medicina, University of Porto, Porto, Portugal; 15 Intensive Care Department, Hospital de Clínicas de Porto Alegre, Porto Alegre, RS, Brazil; 16 UFCSPA Medical School, Porto Alegre, RS, Brazil; Azienda Ospedaliero Universitaria Careggi, ITALY

## Abstract

**Background:**

To mitigate mortality among critically ill COVID-19 patients, both during their Intensive Care Unit (ICU) stay and following ICU discharge, it is crucial to measure its frequency, identify predictors and to establish an appropriate post-ICU follow-up strategy.

**Methods:**

In this multicentre, prospective cohort study, we included 586 critically ill COVID-19 patients.

**Results:**

We observed an overall ICU mortality of 20.1% [95%CI: 17.1% to 23.6%] (118/586) and an overall hospital mortality of 25.4% [95%CI: 22.1% to 29.1%] (149/586). For ICU survivors, 30 days (early) post-ICU mortality was 5.3% [95%CI: 3.6% to 7.8%] (25/468) and one-year (late) post-ICU mortality was 7.9% [95%CI: 5.8% to 10.8%] (37/468). Pre-existing conditions/comorbidities were identified as the main independent predictors of mortality after ICU discharge: hypertension and heart failure were independent predictors of early mortality; and hypertension, chronic kidney disease, chronic obstructive pulmonary disease and cancer were independent predictors of late mortality.

**Conclusion:**

Early and late post-ICU mortality exhibited an initial surge (in the first 30 days post-ICU) followed by a subsequent decline over time. Close monitoring of critically ill COVID-19 post-ICU survivors, especially those with pre-existing conditions, is crucial to prevent adverse outcomes, reduce mortality and to establish an appropriate follow-up strategy.

## Introduction

Critically ill COVID-19 patients present greater need for life-sustaining treatments, high mortality, and prolonged length of intensive care unit (ICU) stay [1]. In-hospital mortality can reach 31% and is expected that as much as 76% of patients will require invasive mechanical ventilation (IMV) [2]. Worst long-term sequelae are also expected for ICU COVID-19 survivors, although the one-year mortality following ICU discharge appears to be influenced more by patient’s comorbidities rather than the severity of COVID-19 pneumonia itself [3].

In relation to the recovery from COVID-19, it is not uncommon to witness the persistence of symptoms for several months following the acquisition of SARS-CoV-2 infection [4]. This condition has been recognized as "post-COVID-19 condition" (PCC) in medical literature [5]. However, the understanding and evidence concerning long-term outcomes in individuals who required critical care due to COVID-19 diagnosis are currently limited. A prospective cohort study conducted in Spain in 2021, evaluating the sequelae occurred at the first month after hospital discharge among patients who required ICU admission for severe COVID-19 pneumonia, concluded that these patients need a long-term follow-up due to its expected bad prognosis, and this should be extended beyond 30 days after ICU discharge [6]. However, evidence regarding the optimal duration for follow-up care in individuals who have survived COVID-19 after being admitted to the ICU is still lacking [7]. The present study aimed to assess early and late mortality after ICU discharge among surviving critical COVID-19 patients and describe their most relevant predictors, in order to propose recommendations regarding the most appropriate follow-up strategy for COVID-19 ICU survivors.

## Materials and methods

### Study design

We conducted a 12-month prospective multicentre cohort study, assessing a large sample of critical COVID-19 patients, admitted in 3 ICU from three tertiary hospitals at North Portugal. Participant hospitals were public and two of them were academic. The recruitment occurred from May 30, 2020, to July 31, 2021. The study included patients admitted to the ICU from March 1st, 2020, to July 31, 2021, and the follow up period ended on August 31, 2022. Medical records were accessed solely for research purposes during the study period, and only the authors had access to information that could potentially identify individual participants. Participant information was included in the study database using unique codes as a means to safeguard all personal data.

All consecutive patients admitted at ICU with COVID-19 infection were recruited after ICU discharge and have been followed-up through structured telephone interviews carried out by trained investigators who were not involved in patient care. These interviews were conducted at regular intervals of 3 months over a 12-month follow-up period with a permissible window of approximately 15 days before or after the scheduled date. After obtaining approval from the institutional review boards (IRB) of all participating centers (Comissão de Ética (CES) do Centro Hospitalar Universitário de São João, Comissão de Ética do Centro Hospitalar Universitário de Santo António, Comissão de Ética do Centro Hospitalar Vila Nova de Gaia/Espinho), patients or their proxies were invited to join the follow up cohort after ICU discharge, and patients’ survival status during follow-up was verified by telephone.

Informed consent for participation in the study was obtained orally from all study participants or their proxies, during the initial telephone contact, due to the pandemic context. IRB of all participant hospitals approved the use of oral consent in the study. The consent was documented, and researchers offered all participants a physical copy of the informed consent form to be sent by postal mail. The research team strongly recommended that patients or their proxies to read and keep a physical copy of the document. Furthermore, investigators reiterated the information that participants could withdraw their consent to participate in the study at any time, during the telephone follow-up, without any adverse consequences.

### Participants

All critical COVID-19 patients aged 18 years or older admitted in the participant ICUs were consecutively enrolled, if they stayed in the ICU for at least 24 hours, regardless of the type of admission to the ICU (medical and surgical, elective or emergency, admissions). The exclusion criteria were as follows: 1) transfer from another ICU at a non-participant hospital; 2) discharge from the ICU to another ICU at a non-participant hospital; 3) previous enrollment in the study; 4) patients unable to communicate and with no proxy; 5) patients refusing or withdrawing their consent and 6) patients with no telephone contact.

### Outcomes

In the present study, the primary outcome was defined as the incidence of all-cause mortality 1 year after ICU discharge (late post-ICU mortality). Secondary outcomes were ICU mortality and early post-ICU mortality, defined as death within 30 days after ICU discharge.

### Data collection

To evaluate the predictors of ICU mortality and post-ICU mortality (early and late), the following variables were assessed and recorded: sociodemographic characteristics, pre-ICU health status and critical illness characteristics. Variables were collected using a structured formulary completed by systematically reviewing the medical records and using a structured questionnaire during the follow-up interviews.

The clinical characterization of COVID-19 infection was strongly recommended by the World Health Organization (WHO) to warrant an appropriate management of COVID-19 patients [8], measuring patient progression through the health-care system, which reflects patient trajectory and resource use over the course of clinical illness. In line with WHO recommendations, the researchers considered the complexity of treatment, which encompassed hospitalization, the use of supplemental oxygen, high-flow nasal cannula oxygen therapy, non-invasive ventilation, and mechanical ventilation (MV) [8].

The Charlson Comorbidity Index (CCI) [9] was used to assess patient’s comorbidities. The index was dichotomized into low comorbidity (score 0 or 1) and high comorbidity (score ≥ 2) categories. The severity of critical illness was assessed based on the Simplified Acute Physiology Score 2 (SAPS 2) [10]. Sepsis and acute respiratory distress syndrome (ARDS) were defined based on medical evaluation and careful examination of clinical records.

Organ disfunction was determined based on the presence of specific conditions and the use of procedures during ICU stay, including the requirement for MV, use of vasopressors, need of renal replacement therapy (RRT) (not considering patients submitted to chronic dialysis treatment), dependence on parenteral nutrition, receipt of blood or blood products transfusion, occurrence of delirium (according to clinical team evaluation) and the development of any ICU-acquired infections, such as pneumonia, bloodstream infection, or urinary tract infection occurring after 48 hours of ICU admission.

During the 12-month follow-up, long-term outcomes were assessed by trained researchers not associated with patient care using structured telephone interviews at three, six, nine and 12 months after ICU discharge. The study was conducted according to the guidelines of the Declaration of Helsinki and approved by the Ethical Committees of every participating hospital. Patients and proxies were informed orally about study procedures and their right to refuse participation at the first telephone contact and a written document about the study was offered to participants and proxies by site investigators to be sent for email or physical correspondence, so a written informed consent was also obtained. If the patient was unable to receive appropriate information, decisions were made by a substitute decision maker, being informed about participation as soon as their clinical status allowed.

### Statistical analysis

Continuous variables are expressed as median and interquartile range (IQR). Categorical variables are expressed as counts and percentages. The normal distribution of variables was evaluated using the Kolmogorov‐Smirnov test. We did not perform imputation for missing data, because the rate of missingness for all variables analyzed was very low (0% for outcome variables and less than 3% for all predictors). Kaplan-Meier curves were used to assess survival after ICU discharge. Logistic regression models were used to assess the association between independent variables and ICU mortality and Cox regression models were used to assess the association between independent variables and early and late post-ICU mortality. All variables with a p value of less than 0.20 in the univariable analysis were included in the multivariable model using the stepwise forward selection method. Results are presented as hazard ratios (HRs) and 95% Cis. The Schoenfeld test was used to verify the validity of the proportionality assumption for Cox regressions (S1 and S2 Figs). Specifically, the association between acute disease severity and post-ICU mortality (early and late) was assessed using Cox regression model (adjusted for age, sex, and number of previous comorbidities). Statistical significance was defined as a p-value less than 0.05 for all comparisons. Analyses were performed with R software (R Development Core Team, Vienna, Austria) [11].

## Results

From May 2020 to July 2021, 601 COVID patients admitted to the ICU were screened and 586 met eligibility criteria and were enrolled in the study. 468 patients survived after ICU admission and were included in the 12-month follow-up cohort. Among the 586 patients enrolled, 155 (26.5%) died before completing 1 year of follow-up and of these, 118 patients (20.1%) died in the ICU and 37 (6.4%) patients died after ICU discharge. Amongst the 468 patients eligible for the 1-year follow-up, which was completed on August 16, 2022, all patients were assessed.

The characteristics of all patients enrolled are summarized in **Table 1**. Median age was 65 years; 32.1% of patients were women. Regarding pre-ICU state of health, 75.9% of patients had high comorbidity and the most common comorbidities were hypertension (61.3%), obesity (35.8%), and diabetes (30.7%). Asthma and chronic obstructive pulmonary disease (COPD) were present in 4.9% and 5.6% of the patients, respectively.

**Table 1:** Characteristics of enrolled patients.

Characteristics	Total
	Patients (n = 586)
**Sociodemographic characteristics**	
Age, years–median (IQR) (n assessed)	65 (57–72)
Age ≥65 years–no./total no. (%)	298/586 (50.9)
Female sex–no./total no. (%)	188/586 (32.1)
**State of health before admission to the ICU**	
Charlson comorbidity index–median (IQR)	3 (2–4)
Charlson comorbidity index ≥2 –no./total no. (%)	445/586 (75.9)
Obesity–no./total no. (%)	210/586 (35.8)
Hypertension–no./total no. (%)	359/586 (61.3)
Diabetes–no./total no. (%)	180/586 (30.7)
Severe CKD–no./total no. (%)	33/586 (5.6)
Dementia–no./total no. (%)	5/586 (0.9)
Asthma–no./total no. (%)	33/586 (5.6)
COPD–no./total no. (%)	29/586 (4.9)
Cancer–no./total no. (%)	33/586 (5.6)
Heart failure–no./total no. (%)	35/586 (6.0)
**Characteristics of acute critical illness**	
** ICU admission type**	
Medical–no./total no. (%)	577/586 (98.5)
Surgical, elective–no./total no. (%)	5/586 (0.9)
Surgical, emergency–no./total no. (%)	4/586 (0.7)
Severity of critical illness at ICU admission (SAPS-2)–median (IQR)	33 (25–42)
Sepsis or septic shock at ICU admission–no./total no. (%)	22/586 (3.8)
** Highest score on six-point ordinal severity scale**	
Score 2: no oxygen therapy–no./total no. (%)	30/586 (5.1)
Score 3: oxygen by mask or nasal prongs–no./total no. (%)	26/586 (4.0)
Score 4: high-flow nasal cannula oxygen therapy or non-invasive ventilation–no./total no. (%)	177/586 (30.2)
Score 5: mechanical ventilation–no./total no. (%)	208/586 (35.5)
Score 6: death (considered until 60 days after hospital discharge)–no./total no. (%)	145/586 (24.7)
** Organ dysfunction during ICU stay**	
Need of invasive mechanical ventilation–no./total no. (%)	316/586 (53.9)
Days of mechanical ventilation—median (IQR)	13 (8–23)
Need of noninvasive ventilation–no./total no. (%)	181/586 (30.9)
Days of noninvasive ventilation—median (IQR)	3 (1–5.5)
Need of oxygen therapy–no./total no. (%)	458/586 (78.2)
Days of oxygen therapy—median (IQR)	4 (2–7)
Need of high flow oxygen therapy—no./total no. (%)	373/586 (63.7)
Need of vasopressor–no./total no. (%)	301/586 (51.4)
Need of RRT- no./total no. (%)	56/586 (9.6)
Need of parenteral nutrition–no./total no. (%)	12/586 (2.0)
Need of blood or blood products transfusion–no./total no. (%)	95/586 (16.2)
Delirium–no./total no. (%)	115/586 (19.6)
Any ICU-acquired infection–no./total no. (%)	255/586 (43.5)
Bacterial pneumonia–no./total no. (%)	225/586 (38.4)
Bloodstream infection–no./total no. (%)	109/586 (18.6)
Urinary tract infection–no./total no. (%)	62/586 (10.6)
Treatment limitations–no./total no. (%)	69/586 (11.8)
ICU length of stay, days, median (IQR)	9 (3–18)
Hospital length of stay, days, median (IQR)	18 (10–35)

IQR—Interquartile Range, ICU–Intensive Care Unit, CKD—chronic kidney disease, COPD—Chronic obstructive pulmonary disease, RRT—Renal Replacement Therapy.

Regarding critical illness, 1.2% of patients were admitted to the ICU due to non-medical (surgical) reasons. At ICU admission, the median SAPS-2 score was 33. According to the highest score on the WHO ordinal severity scale during hospital stay [8], 30 (5.1%) were categorized as score 2, 26 (4.0%) as score 3, 177 (30.2%) as score 4, 208 (35.5%) as score 5, and 145 (24.7%) as score 6. Regarding organ dysfunctions during ICU stay, 30.9% of patients needed non-invasive oxygen therapy, 53.9% of patients needed MV and 51.4% needed vasopressors. Less than 10% needed RRT and delirium was diagnosed in 19.6%. The median length of ICU and hospital stay were, respectively, 9 and 18 days, and the incidence of any ICU-acquired infections was 43,5%, mostly bacterial pneumonia (38.4%). Almost 12% of patients had treatment limitations justified by clinical decision during ICU stay.

### Mortality

At the time of ICU discharge, health status was evaluated as “worse than it was before ICU admission” by 88.9% of the patients and 1.3% were directly discharged home after ICU. Overall ICU mortality was 20,1% [95%CI: 17.1% to 23.6%] (118 of 586 critically ill COVID-19 patients) and overall hospital mortality was 25.4% [95%CI: 22.1% to 29.1%] (149 of 586 critically ill COVID-19 patients).

We considered as early mortality all deaths that occurred during the first 30 days after ICU discharge, and we observed a 5.3% [95%CI: 3.6% to 7.8%] (25/468) early post-ICU mortality rate, among ICU survivors. Late mortality was defined as deaths occurring during the first 365 days after ICU discharge. Between days 31st and 365th after ICU discharge, we observed a 2.7% [95%CI: 1.6% to 4.7%] late post-ICU mortality rate, among ICU survivors that were alive at 30 days after ICU discharge (12/439). Thus, we observed an overall mortality rate among ICU survivors during the first year after ICU discharge of 7.9% [95%CI: 5.8% to 10.8%] (37/468) and an overall 1-year mortality among all critically ill COVID-19 patients of 26.5% [95%CI: 23.0% to 30.2%] (155/586). Among the 37 patients who died after ICU discharge, 31 patients died in-hospital (from these, 25 patients died up to 30 days and 6 died between 31 and 365 days after ICU discharge) and 6 patients died after hospital discharge.

**Fig 1:**
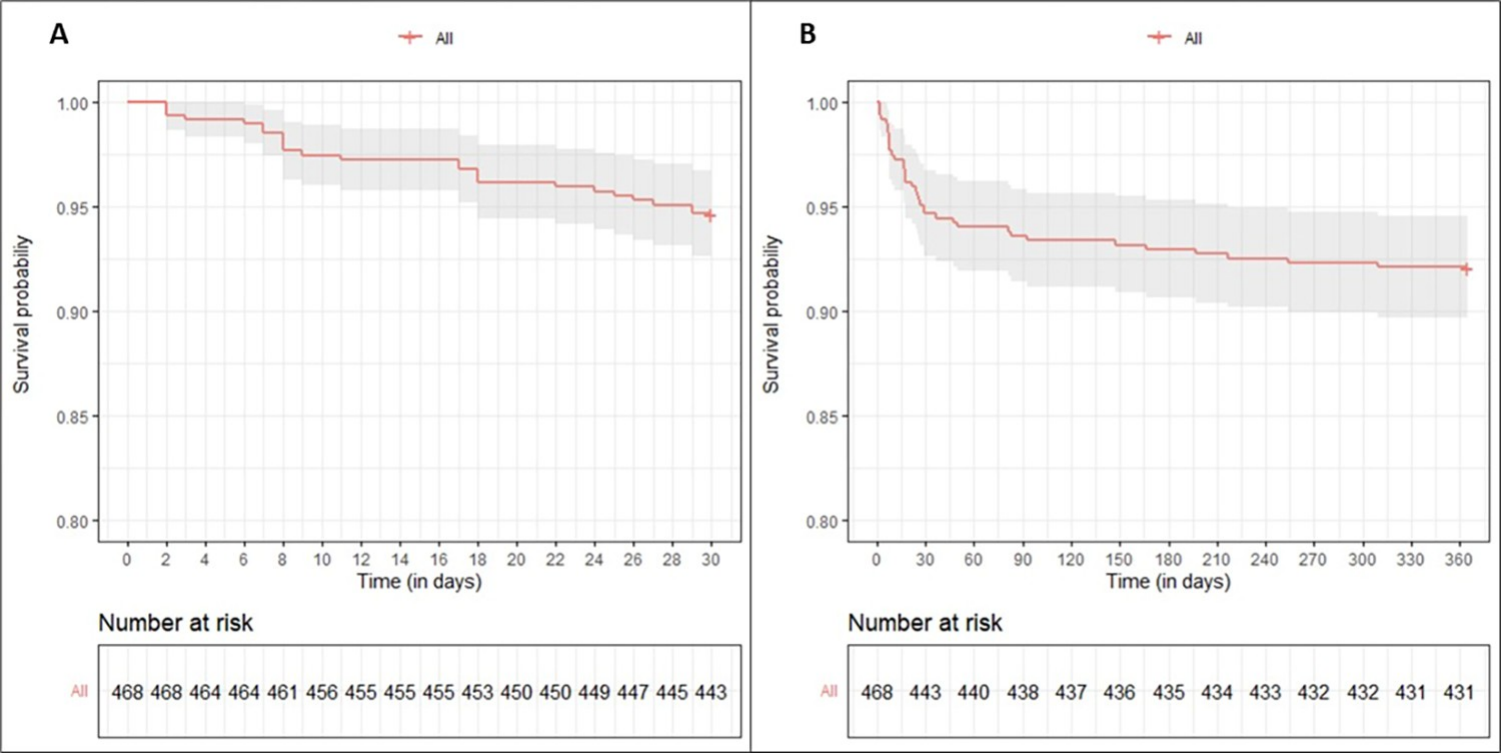
ICU survivors data represented as Kaplan-Meier survival curves. *Caption*: **A,** Survival curve of the 30 days after ICU discharge. **B,** Survival curve during the one-year follow up.

Regarding all COVID-19 patients admitted to the ICU, one-year mortality was 15.9% [95%CI: 11.6% to 20.3%] and 35.4% [95%CI: 30.2% to 40.7%] in non-invasive ventilated patients (43 of 270 patients) and patients submitted to IMV (112 of 316 patients), respectively. Fig 1 shows survival data over time. Median time to death was 11 days after ICU discharge (IQR, 7.0–23.0) for early mortality, and 22 days after ICU discharge (IQR, 8.0–65.5) for all patients dying during the first year after ICU discharge.

### Predictors of ICU mortality and early and late post-ICU mortality

Results of univariable analysis for predictors of ICU mortality and multivariable analysis for predictors independently associated with ICU mortality are shown in S1 and S2 Tables, respectively. Severity of critical illness, age greater than or equal to 65 years, delirium, ICU-acquired bacterial pneumonia, need of RRT, oxygen therapy, vasopressor, and the length of ICU stay were associated with ICU mortality.

Results of univariable analysis for predictors of early and late post-ICU mortality are presented in Table 2 and multivariable analysis for predictors independently associated with early and late post-ICU mortality are presented in Table 3. Detailed univariable analysis for predictors of early and late post-ICU mortality can be found in S3 and S4 Tables, respectively. Previously diagnosed conditions (comorbidities) were identified as the most relevant and significant independent predictors associated with early and late mortality after ICU discharge. Pre-ICU hypertension (HR, 5.24; p = 0.007) and pre-ICU heart failure (HR, 4.74; p = 0.002) were independent predictors associated with early post-ICU mortality. Pre-ICU hypertension (HR, 4.96; p < 0.001), pre-ICU chronic kidney disease (CKD) (HR, 1.84; p = 0.007), pre-ICU chronic obstructive pulmonary disease (HR, 4.62; p = 0.018) and pre-ICU cancer (HR, 1.79; p = 0.017) were the independent predictors associated with late post-ICU mortality.

**Table 2:** Univariable analysis of predictors associated with early (up to 30 days after ICU discharge) and late mortality (up to 365 days after ICU discharge).

	Early Mortality		Late Mortality	
Characteristics	Hazard ratio (95%CI)	*P*-value	Hazard ratio (95%CI)	*P*-value
Sociodemographic				
Age, years–median (IQR)	1.70 (1.03–1.11)	<0.001	1.05 (1.02–1.08)	0.001
Age ≥65 years–no./total no. (%)	3.77 (1.11–12.81)	0.033	1.57 (0.72–3.45)	0.26
Female sex–no./total no. (%)	1.18 (0.52–2.67)	0.693	1.03 (0.51–2.05)	0.941
Pre-ICU state of health				
Charlson comorbidity index–median (IQR)	1.25 (1.09–1.44)	0.001	1.28 (1.15–1.43)	<0.001
High comorbidity^a^ –no./total no. (%)	0.73 (0.16–3.28)	0.735	1.84 (0.61–5.59)	0.281
Comorbidities				
Hypertension–no./total no. (%)	5.24 (1.52–18.0)	0.009	3.90 (1.63–9.35)	0.002
Obesity–no./total no. (%)	0.85 (0.26–1.98)	0.708	1.02 (0.52–2.03)	0.945
Diabetes–no./total no. (%)	0.98 (0.43–2.22)	0.953	1.19 (0.60–2.37)	0.614
Asthma–no./total no. (%)	0.85 (0.11–6.31)	0.874	0.52 (0.07–3.80)	0.52
Cancer–no./total no. (%)	1.12 (0.26–4.89)	0.879	2.26 (0.85–6.00)	0.1
Chronic obstructive pulmonary disease–no./total no. (%)	1.04 (0.14–7.69)	0.97	2.46 (0.75–8.09)	0.139
Heart failure–no./total no. (%)	5.16 (1.91–13.95)	0.001	3.73 (1.52–9.12)	0.004
Chronic kidney disease–no./total no. (%)	2.25 (0.67–7.63)	0.191	4.08 (1.61–10.30)	0.003
History of a cerebrovascular accident–no./total no. (%)	0	0.977	0.96 (0.23–4.02)	0.951
Critical illness				
Severity of critical illness at ICU admission^b^,–median (IQR)	1.00 (0.97–1.03)	0.856	1.00 (0.97–1.02)	0.77
Sepsis or septic shock at ICU admission–no./total no. (%)	1.74 (0.24–1.98)	0.587	2.39 (0.57–9.93)	0.232
Organ dysfunctions during ICU stay				
Delirium–no./total no (%)	0.67 (0.23–1.95)	0.461	1.12 (0.53–2.38)	0.761
Need of non-invasive mechanical ventilation–no./total no. (%)	0.94 (0.37–2.40)	0.9	0.69 (0.31–1.53)	0.364
Need of low-flow oxygen therapy–no./total no. (%)	0.59 (0.23–1.48)	0.258	0.43 (0.20–0.93)	0.43
Need of high-flow oxygen therapy–no./total no. (%)	0.61 (0.26–1.43)	0.609	0.55 (0.28–1.09)	0.085
Need of invasive mechanical ventilation–no./total no. (%)	0.44 (0.05–4.15)	0.473	0.93 (0.47–1.83)	0.83
Need of vasopressor–no./total no. (%)	1.79 (0.20–16.40)	0.606	0.85 (0.44–1.63)	0.623
Need of renal replacement therapy–no./total no. (%)	0.64 (0.09–4.73)	0.663	1.37 (0.42–4.46)	0.602
Need of blood or blood products transfusion–no./total no. (%)	-	0.972	0.83 (0.29–2.34)	0.721
Need of parenteral nutrition–no./total no. (%)	-	0.991	2.41 (0.31–18.76)	0.399
Length of ICU stay, days–median (IQR)	0.99 (0.96–1.02)	0.499	1.0 (0.97–1.02)	0.762
Any-ICU acquired infections^c^ –no./total no. (%)	0.72 (0.21–2.44)	0.595	0.53 (0.17–1.61)	0.264
Pneumonia–no./total no. (%)	2.14 (0.69–6.64)	0.073	1.62 (0.66–3.98)	0.288
Bloodstream infection–no./total no. (%)	2.14 (0.69–6.64)	0.186	2.06 (0.76–5.58)	0.154

CI—confidence interval; ICU—intensive care unit; IQR—interquartile range (p25-p75).

^a^ Charlson comorbidity index ≥2.

^b^ The severity of critical illness at ICU admission was calculated using established prediction equations for hospital death according to the Simplified Acute Physiology Score-2.

^c^ Pneumonia, bloodstream infection, or urinary tract infection according to the European Centre for Disease Prevention and Control criteria.

**Table 3:** Multivariable regression models with independent predictors for early and late post-ICU mortality.

Variable	Hazard Ratio^a^ (95% CI)	P
**Early post-ICU mortality (up to 30 days after ICU discharge)**		
Pre-ICU Hypertension	5.24 (1.56–17.52)	0.007
Pre-ICU heart failure	4.74 (1.78–12.66)	0.002
**Late post-ICU mortality (up to 365 days after ICU discharge)**		
Pre-ICU Hypertension	4.96 (1.92–12.80)	<0.001
Pre-ICU chronic kidney disease	1.84 (1.18–2.88)	0.007
Pre-ICU chronic obstructive pulmonary disease	4.62 (1.30–16.37)	0.018
Pre-ICU cancer	1.79 (1.11–2,90)	0.017

^a^ The hazard ratio (HR) was calculated using Cox Regression for early and late mortality.

## Discussion

In the present cohort study, performed in adult critically ill patients with COVID-19 admitted to the ICU and followed up during the first 12 months after ICU discharge, we observed an ICU mortality of 20.1%, which is inferior to previous studies where the reported mortality ranged between 30 to 65% [12–14]. Moreover, regarding ICU survivors, early mortality, considering 30 days after ICU discharge, was 5.3% and late mortality (from day 31 to day 365) was 2.6%; thus, the overall 1-year mortality observed among ICU survivors was 7.9% and the overall 1-year mortality among all the COVID-19 patients admitted to the ICU was 26.5%.

There are very few studies reporting the long-term outcomes of critically ill COVID-19 patients. Those studies have shown a long-term mortality (at 1-year follow up) for COVID-19 survivors between 3.5% and 7.5% [3, 15], and these studies rarely report separate estimates for early and late mortality. In the present study, both early and late ICU mortality were higher in patients with more severe COVID-19 illness, defined as need for MV during hospitalisation, which is consistent with the reports from previous studies and a systematic review by Serafim et al. [1]. Despite MV is a necessary treatment for most severely ill COVID-19 patients, it has been associated with adverse long-term outcomes, including hospital readmission and death [16].

We observed that advanced age, SAPS 2 score, delirium during ICU, ICU-acquired bacterial pneumonia and the severity/complexity of management of critical disease (need for RRT, oxygen therapy and vasopressors) were associated with ICU mortality for COVID-19 patients. This is consistent with previous findings from observational studies showing that COVID-19 patients who died at ICU tended to be older, experienced more severe disease, had a greater burden of comorbidities, and developed more complications during their ICU stay [17, 18].

We found a 30-day mortality of all COVID-19 patients admitted to the ICU of 24.4%. This is an estimate higher than those reported in some previous studies, for example, a retrospective cohort study involving 2406 critically ill patients aged 70 years and older, that reported mortality rates ranging from 14% to 17% [19], but it is in agreement with a Swedish cohort study, with 6350 adult COVID ICU patients admitted between March 2020 and April 2021, showing a 30-day mortality of 24.3% [20]. Most of all early mortality studies considered all COVID patients admitted to the ICU and not only ICU survivors. Regarding ICU survivors, it is expected an early mortality rate near to 8% for non-COVID ICU patients [21], a little higher than our COVID-19 ICU survivors early mortality rate (5.3%).

In the present cohort, considering all COVID-19 patients admitted to the ICU, one-year mortality (26.5%) was similar to other studies, close to 30% [3, 22]. Late mortality observed in our study for ICU survivors (7.9%) was higher than previous studies from Europe and Latin America, that showed late mortality rates among COVID-19 ICU survivors lower than 5% [3, 22, 23]. Further studies focusing on long-term health status of ICU-survivors are warranted to better explore long-term mortality among ICU COVID-19 survivors, but these results should draw our attention to the need to maintain a closer monitoring during the initial phase after ICU discharge. This same recommendation emerged from previous studies on the general ICU population, showing an early phase after ICU discharge characterized by high mortality rates, followed by a gradual decrease in the risk of death in a 60-day period post-ICU discharge [21].

In our study, 35.4% of COVID-19 patients that received IMV at the ICU died during the first year after ICU admission, data not much lower than that reported [22]. Several potential explanations can be explored to elucidate the correlation between the requirement of MV and unfavorable long-term prognosis among individuals who have survived COVID-19. As previously observed, comparing with other causes of pneumonia, it can be inferred that COVID-19 pneumonia has a lower late mortality rate among ICU survivors [24]. The cause behind reduced long-term mortality rates for COVID patients remains unclear, as the incidence of pulmonary anatomical abnormalities and compromised physiological function is significantly high among individuals diagnosed with ARDS as a consequence of COVID-19 [6]. Additionally, research findings indicate that COVID-19 patients necessitating MV exhibit heightened levels of inflammatory markers, multiple organ dysfunction, and increased rates of in-hospital mortality [24–26]. These findings may underscore the influence of pre-existing health conditions on adverse outcomes among survivors of severe COVID-19 cases.

Regarding predictors of post-ICU mortality, pre-ICU comorbidities were the only factors associated with mortality after ICU discharge. Similarly, in our study, previous heart failure increased early mortality and previous severe CKD, COPD and cancer, increased late mortality. Our findings are aligned with prior studies, indicating that the presence of pre-existing comorbidities has a greater impact on post-discharge mortality than the severity of COVID-19 pneumonia itself [3, 15, 27, 28]. Other cohort studies also showed that previous hypertension and CKD increased both early and late mortality [29–31]. Previous studies have consistently shown a significantly higher risk of in-hospital deaths, ICU admissions, and the need for invasive ventilation among individuals with a combination of COVID-19 infection and hypertension [32]. It has been shown that COVID-19 patients admitted to the ICU had a higher comorbidity burden than those not admitted to the ICU [33].

Our data support that the burden directly attributable to COVID-19 in terms of hospitalisation and mortality is notably elevated, surpassing the impact attributed to acute illness alone. Clinicians should develop an effective post-discharge follow-up plan for these patients, considering the factors that may contribute to mortality after discharge.

Our study has several strengths, including the examination of both the ICU period and the post-discharge follow-up period, which provides valuable insights into the planning and management of post-ICU care [34]. In addition, it is a prospective cohort with the assessment of patient-centered outcomes contributing to the standardization of eligibility criteria and data collection methods. This approach not only facilitated consistent data collection but also minimized the occurrence of missing values for fundamental variables. A multicentre design and interviews conducted by trained personnel unrelated to the patient’s care appeared to be successful to avoid potential bias.

Some limitations must be considered. First, the inclusion of patients during the first wave of the pandemic, when the healthcare system was overwhelmed and still in the process of organization may have resulted in some missing data; however, it has been shown that there were no significant differences in ICU mortality observed between waves [17]. Second, COVID-19 can have different effects on long-term outcomes in different contexts as a consequence of differences in post-discharge access to health services and we have not considered these aspects in the present study. Particularly, our study did not evaluate treatment limitations imposed following ICU discharge, which could potentially be significant factors contributing to post-ICU mortality. Third, it is important to note that the present study includes only patients enrolled during the first 14 months of the COVID-19 pandemic, which may limit the generalizability of the results, especially considering the substantial changes in the course of the pandemic over time, mostly after the Omicron variant became dominant [35]. However, we should also highlight the importance of the rigorous analysis of the initial phase of the pandemic and the many relevant lessons learned from this, as most of the predictors of long-term outcomes in critical care described and discussed are applicable and generalizable in broader contexts. Finally, we did not assess variables that have the potential to modify the association between the acute severity of COVID-19 and long-term outcomes, such as vaccination status and infection by different variants of the virus.

## Conclusion

By reporting on the early and late mortality post-ICU discharge in critically ill COVID-19 patients, we have shown that there is a period immediately following ICU discharge, specifically in the first 30 days, with a higher mortality rate, but this risk decreases over time up to 1 year. Therefore, it is crucial to closely monitor critically ill COVID-19 patients who have survived intensive care, particularly those with pre-existing comorbidities, to prevent adverse outcomes and mortality, as our findings indicate that pre-ICU comorbidities were the sole factors associated with mortality after ICU discharge. Since we identified a limited number of previous studies, further research is urgently needed on post-ICU mortality rates and to establish an appropriate post-ICU follow-up strategy for surviving critically ill COVID-19 patients.

## Supporting information

S1 Fig Schoenfeld test for the early post-ICU mortality multivariable Cox regression model. (TIF)

S2 Fig Schoenfeld test for the late post-ICU mortality multivariable Cox regression model. (TIF)

S1 Table Univariable analysis of predictors associated with ICU mortality. (PDF)

S2 Table Multivariable analysis of predictors associated with ICU mortality. (PDF)

S3 Table Univariable analysis of predictors associated with early post-ICU mortality. (PDF)

S4 Table Univariable analysis of predictors associated with late post-ICU mortality. (PDF)
