# Supplementary figures and images for: Predictors of early and long-term mortality after ICU discharge in critically ill COVID-19 patients: A prospective cohort study
Source: PLoS One. 2023 Nov 2;18(11):e0293883. doi: 10.1371/journal.pone.0293883 (PMC10621933; doi:10.1371/journal.pone.0293883)

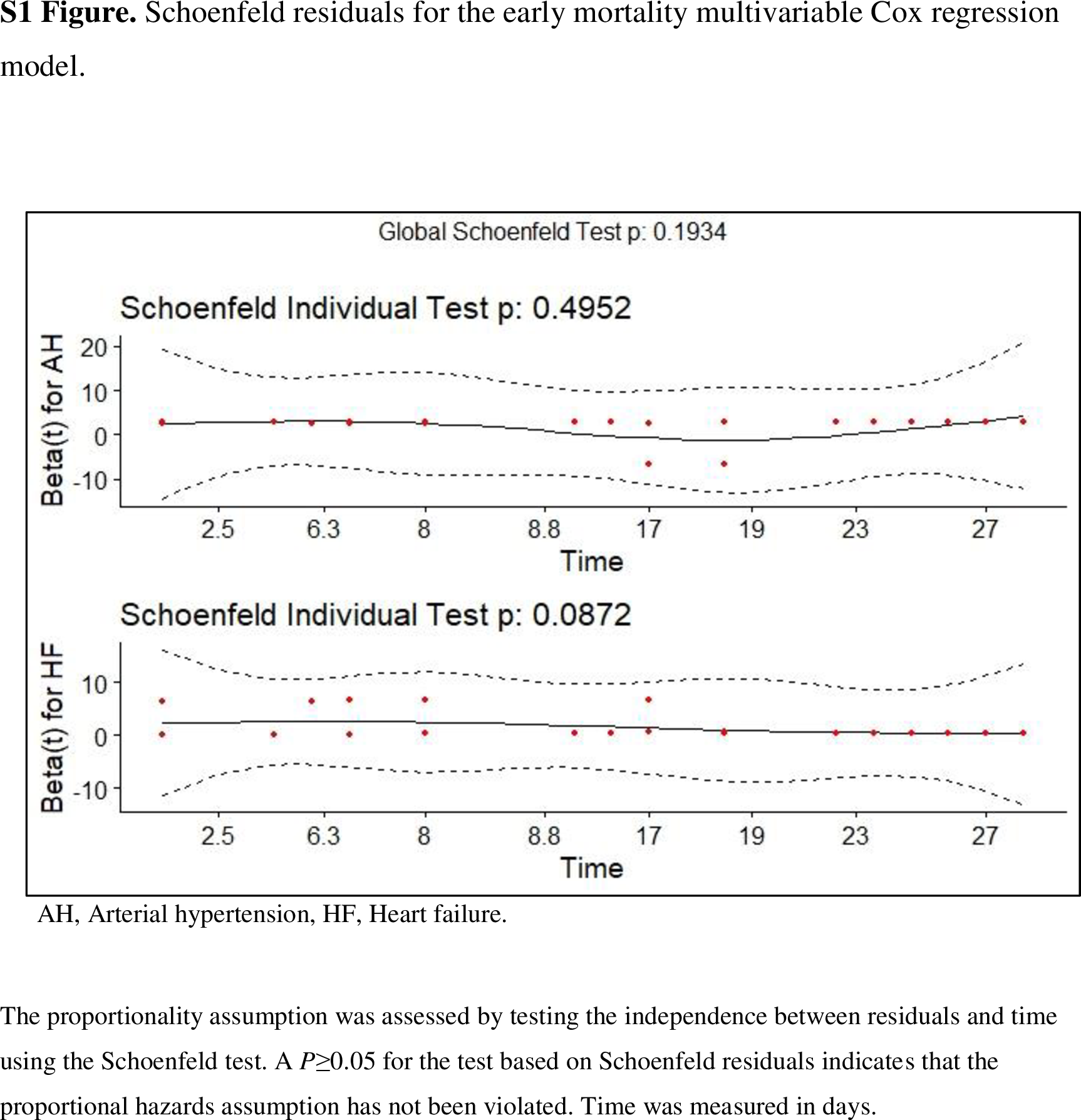

Supplement: S1 Fig — (TIF) [file pone.0293883.s001.tif]

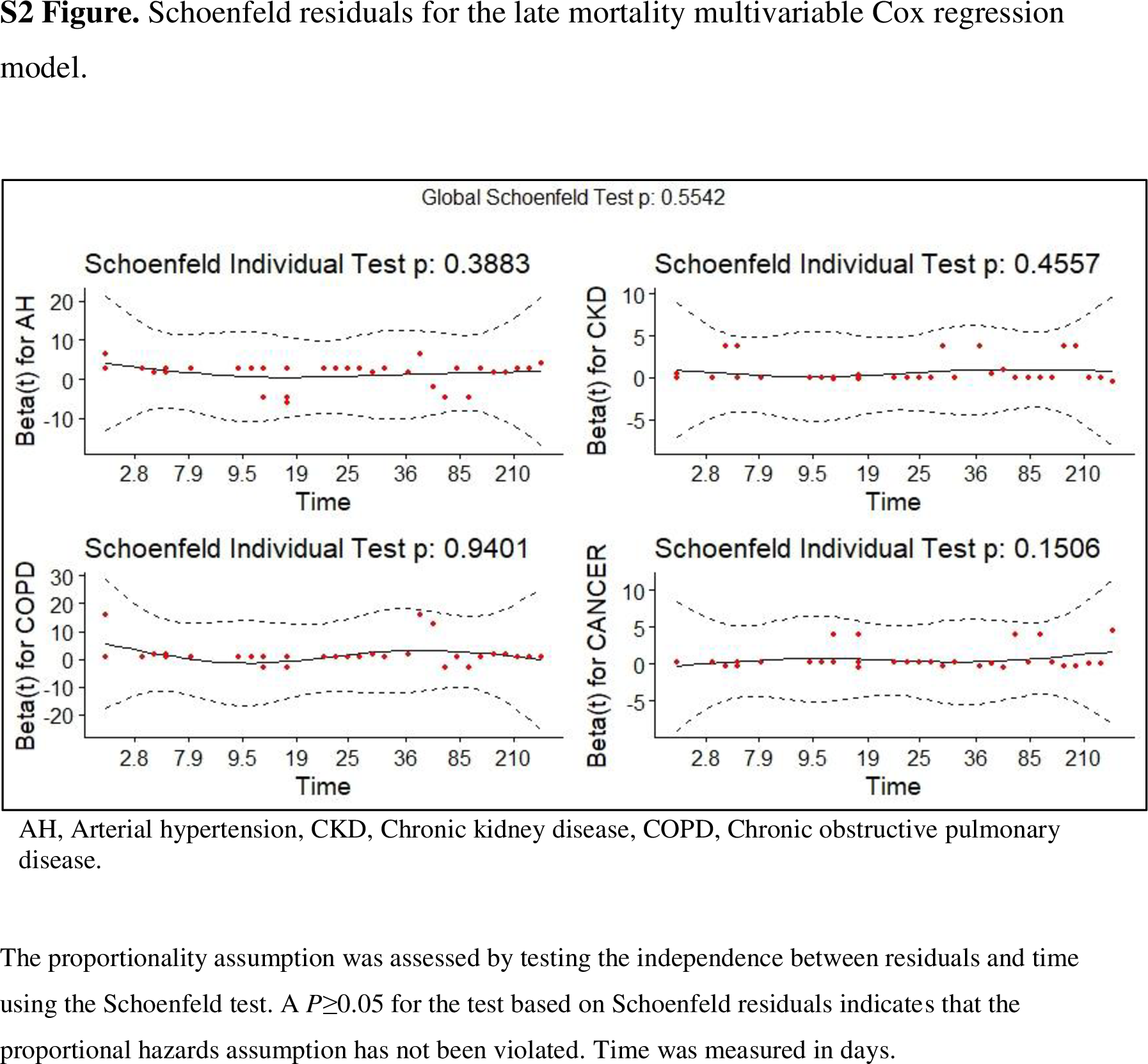

Supplement: S2 Fig — (TIF) [file pone.0293883.s002.tif]
