# Supplementary material for: Predictors of early and long-term mortality after ICU discharge in critically ill COVID-19 patients: A prospective cohort study
Source: PLoS One. 2023 Nov 2;18(11):e0293883. doi: 10.1371/journal.pone.0293883 (PMC10621933; doi:10.1371/journal.pone.0293883)
Supplement: S1 Table — (PDF) [file pone.0293883.s003.pdf]

S1 Table. Univariable analysis of factors associated with ICU mortality.

Characteristics	Mortality group (no.=118)	Survival group (no.=468)	Hazard ratio (95%CI)	*P*-value
Sociodemographic				
Age, years – median (IQR)	70.0 (63.0-75.2)	63.0 (55.0-71.0)	1.06 (1.04 - 1.08)	<0.001
Age ≥65 years – no./total no. (%)	85/118 (72.0)	213/468 (45.5)	3.08 (1.98 - 4.79)	<0.001
Female sex – no./total no. (%)	33/118 (28.0)	155/468 (33.1)	0.78 (0.50 - 1.22)	0.285
Pre-ICU state of health				
Charlson comorbidity index – median (IQR)	4.0 (2.0-5.0)	3.0 (1.0-4.0)	1.22 (1.12 - 1.34)	<0.001
High comorbidity[a] – no./total no. (%)	104/118 (88.1)	341/468 (72.9)	2.77 (1.53 - 5.01)	<0.001
Comorbidities				
Hypertension – no./total no. (%)	88/118 (74.6)	271/468 (57.9)	2.14 (1.36 - 3.63)	0.001
Obesity – no./total no. (%)	38/118 (32.2)	172/468 (36.8)	0.82 (0.53 - 1.26)	0.358
Diabetes – no./total no. (%)	36/118 (30.5)	144/468 (30.8)	0.99 (-0.64 - 1.53)	0.956
Asthma – no./total no. (%)	7/118 (5.9)	26/468 (5.6)	1.13 (0.47 - 2.7)	0.782
Cancer – no./total no. (%)	7/118 (5.9)	26/468 (5.6)	1.07 (0.45 - 2.53)	0.874
Chronic obstructive pulmonary disease – no./total no. (%)	9/118 (7.6)	20/468 (4.3)	1.88 (0.83 - 4.27)	0.129
Heart failure – no./total no. (%)	11/118 (9.3)	24/468 (5.1)	1.90 (0.90 - 4.0)	0.09
Chronic renal disease – no./total no. (%)	12/118 (10.2)	21/468 (4.5)	2.55 (1.21 - 5.39)	0.014
History of a cerebrovascular accident – no./total no. (%)	11/118 (9.3)	26/468 (5.6)	1.75 (0.84 - 3.65)	0.137
Critical illness				

Risk of death at ICU admission[b], % – median (IQR)	40.0 (32.2-53.8)	31.0 (24.0-39.0)	1.05 (1.04 - 1.07)	<0.001
Sepsis or septic shock at ICU admission – no./total no. (%)	11/118 (9.3)	11/468 (2.4)	4.27 (1.80 - 10.11)	<0.001
Organ dysfunctions during ICU stay				
Delirium – no./total no (%)	13/118 (11.0)	102/468 (21.8)	0.44 (0.24 - 0.82)	0.01
Need of non-invasive mechanical ventilation – no./total no. (%)	33/118 (28.0)	148/468 (31.6)	0.84 (0.54 - 1.31)	0.443
Need of low-flow oxygen therapy – no./total no. (%)	6/118 (5.1)	176/468 (37.6)	0.09 (0.04 - 0.21)	<0.001
Need of high-flow oxygen therapy – no./total no. (%)	47/118 (39.8)	326/468 (69.7)	0.29 (0.19 - 0.44)	<0.001
Need of invasive mechanical ventilation – no./total no. (%)	96/118 (81.4)	220/468 (47.0)	4.92 (2.99 - 8.09)	<0.001
Need of vasopressor – no./total no. (%)	95/118 (80.5)	206/468 (44.0)	5.25 (3.22 - 8.58)	<0.001
Need of renal replacement therapy – no./total no. (%)	28/118 (23.7)	28/468 (6.0)	4.89 (2.76 - 8.65)	<0.001
Need of blood or blood products transfusion – no./total no. (%)	37/118 (31.4)	58/468 (12.4)	3.23 (2.01 - 5.2)	<0.001
Need of parenteral nutrition – no./total no. (%)	5/118 (4.2)	7/468 (1.5)	2.91 (0.91 - 9.35)	0.072
Length of ICU stay, days – median (IQR)	13.0 (5.0-20.2)	8.0 (3.0-17.0)	1.01 (1.00 - 1.02)	0.140
Any-ICU acquired infections[c] – no./total no. (%)	82/118 (69.5)	175/468 (37.4)	3.81 (2.47 - 5.89)	<0.001
Pneumonia – no./total no. (%)	69/118 (58.5)	156/468 (33.3)	2.62 (1.70 - 4.03)	<0.001
Bloodstream infection – no./total no. (%)	32/118 (27.1)	77/468 (16.5)	1.37 (0.83 - 2.27)	0.212

CI, confidence interval; ICU, intensive care unit; IQR, interquartile range (p25-p75).

[a] Charlson comorbidity index ≥2.

[b] The risk of death was calculated using established prediction equations for hospital death according to the Simplified Acute Physiology Score-2.

[c] Pneumonia, bloodstream infection, or urinary tract infection according to the European Centre for Disease Prevention and Control criteria.
