# Supplementary material for: Predictors of early and long-term mortality after ICU discharge in critically ill COVID-19 patients: A prospective cohort study
Source: PLoS One. 2023 Nov 2;18(11):e0293883. doi: 10.1371/journal.pone.0293883 (PMC10621933; doi:10.1371/journal.pone.0293883)
Supplement: S2 Table — (PDF) [file pone.0293883.s004.pdf]

S2 Table. Multivariable analysis of factors associated with ICU mortality.

**Variable**	**Odds Ratio[a] (95% CI)**	**P**
**ICU mortality**		
Risk of death at ICU admission	1.02 (1.00 – 1.04)	0.016
Age ≥ 65 years	2.46 (1.46 – 4.50)	0.001
Delirium during ICU stay	0.24 (0.11 – 0.51)	<0.001
ICU aquired bacterial pneumonia	2.05 (1.12 – 3.77)	0.021
Need of renal replacement therapy at the ICU stay	3.19 (1.51 – 6.75)	0.002
Need of low-flow oxygen therapy at the ICUstay	0.06 (0.02 – 0.16)	<0.001
Need of high-flow oxygen therapy at the ICU stay	0.23 (0.13 – 0.40)	<0.001
Need of vasopressors at the ICU stay	3.05 (1.54 – 6.03)	0.001
Length of ICU stay	0.96 (0.93 – 0.99)	0.004

[a] The odds ratio (OR) was calculated using Logistic Binomial Regression.
