# Supplementary material for: Predictors of early and long-term mortality after ICU discharge in critically ill COVID-19 patients: A prospective cohort study
Source: PLoS One. 2023 Nov 2;18(11):e0293883. doi: 10.1371/journal.pone.0293883 (PMC10621933; doi:10.1371/journal.pone.0293883)
Supplement: S3 Table — (PDF) [file pone.0293883.s005.pdf]

S3 Table. Univariable analysis of predictors associated with early post-ICU mortality (up to 30 days after ICU discharge).

Characteristics	Mortality group (no.=25)	Survival group (no.=443)	Hazard ratio (95%CI)	*P*-value
Sociodemographic				
Age, years – median (IQR)	71.0 (59.0-80.0)	63.0 (54.0-70.0)	1.70 (1.03 - 1.11)	<0.001
Age ≥65 years – no./total no. (%)	18/25 (72.0)	195/443 (44.0)	3.77 (1.11 - 12.81)	0.033
Female sex – no./total no. (%)	9/25 (36.0)	146/443 (33.0)	1.18 (0.52 - 2.67)	0.693
Pre-ICU state of health				
Charlson comorbidity index – median (IQR)	4.0 (3.0-5.0)	3.0 (1.0-4.0)	1.25 (1.09 - 1.44)	0.001
High comorbidity[a] – no./total no. (%)	21/25 (84.0)	320/443 (72.2)	0.73 (0.16 - 3.28)	0.735
Comorbidities				
Hypertension – no./total no. (%)	22/25 (88.0)	249/443 (56.2)	5.24 (1.52 - 18.0)	0.009
Obesity – no./total no. (%)	8/25 (32.0)	164/443 (37.0)	0.85 (0.26 - 1.98)	0.708
Diabetes – no./total no. (%)	10/25 (40.0)	134/443 (30.2)	0.98 (0.43 - 2.22)	0.953
Asthma – no./total no. (%)	1/25 (4.0)	25/443 (5.6)	0.85 (0.11 - 6.31)	0.874
Cancer – no./total no. (%)	2/25 (8.0)	24/443 (5.4)	1.12 (0.26 - 4.89)	0.879
Chronic obstructive pulmonary disease – no./total no. (%)	1/25 (4.0)	19/443 (4.3)	1.04 (0.14 - 7.69)	0.97
Heart failure – no./total no. (%)	5/25 (20.0)	19/443 (4.3)	5.16 (1.91 - 13.95)	0.001
Chronic renal disease – no./total no. (%)	3/25 (12.0)	18/443 (4.1)	2.25 (0.67 - 7.63)	0.191
History of a cerebrovascular accident – no./total no. (%)	0/25	26/443 (5.9)	0	0.977

Critical illness				
Risk of death at ICU admission[b], – median (IQR)	32.0 (24.2-37.5)	31.0 (24.0-39.5)	1.00 (0.97 - 1.03)	0.856
Sepsis or septic shock at ICU admission – no./total no. (%)	1/25 (4.0)	10/443 (2.3)	1.74 (0.24 - 1.98)	0.587
Organ dysfunctions during ICU stay				
Delirium – no./total no (%)	4/25 (16.0)	98/443 (22.1)	0.67 (0.23 - 1.95)	0.461
Need of non-invasive mechanical ventilation – no./total no. (%)	7/25 (28.0)	141/443 (31.8)	0.94 (0.37 - 2.40)	0.9
Need of low-flow oxygen therapy – no./total no. (%)	7/25 (28.0)	169/443 (38.1)	0.59 (0.23 - 1.48)	0.258
Need of high-flow oxygen therapy – no./total no. (%)	15/25 (60.0)	311/443 (70.2)	0.61 (0.26 - 1.43)	0.609
Need of invasive mechanical ventilation – no./total no. (%)	10/25 (40.0)	210/443 (47.4)	0.44 (0.05 - 4.15)	0.473
Need of vasopressor – no./total no. (%)	10/25 (40.0)	196/443 (44.2)	1.79 (0.20 - 16.40)	0.606
Need of renal replacement therapy – no./total no. (%)	1/25 (4.0)	27/443 (6.1)	0.64 (0.09 - 4.73)	0.663
Need of blood or blood products transfusion – no./total no. (%)	0/25	58/443 (13.1)	-	0.972
Need of parenteral nutrition – no./total no. (%)	0/25	7/443 (1.6)	-	0.991
Length of ICU stay, days – median (IQR)	8.0 (2.0-18.0)	8.0 (3.0-17.0)	0.99 (0.96 - 1.02)	0.499
Any-ICU acquired infections[c] – no./total no. (%)	11/25 (44.0)	164/443 (37.0)	0.72 (0.21 - 2.44)	0.595
Pneumonia – no./total no. (%)	12/25 (48.0)	144/443 (32.5)	2.14 (0.69 - 6.64)	0.073
Bloodstream infection – no./total no. (%)	6/25 (24.0)	71/443 (16.0)	2.14 (0.69 - 6.64)	0.186

CI, confidence interval; ICU, intensive care unit; IQR, interquartile range (p25-p75).

[a] Charlson comorbidity index ≥2.

[b] The risk of death was calculated using established prediction equations for hospital death according to the Simplified Acute Physiology Score-2.

[c] Pneumonia, bloodstream infection, or urinary tract infection according to the European Centre for Disease Prevention and Control criteria.
