# Supplementary material for: Predictors of early and long-term mortality after ICU discharge in critically ill COVID-19 patients: A prospective cohort study
Source: PLoS One. 2023 Nov 2;18(11):e0293883. doi: 10.1371/journal.pone.0293883 (PMC10621933; doi:10.1371/journal.pone.0293883)
Supplement: S4 Table — (PDF) [file pone.0293883.s006.pdf]

S4 Table. Univariable analysis of predictors associated with late post-ICU mortality (up to 365 days after ICU discharge).

Characteristics	Mortality group (no.=37)	Survival group (no.=431)	Hazard ratio (95%CI)	*P*-value
Sociodemographic				
Age, years – median (IQR)	70.0 (59.0-78.5)	63.0 (54.0-70.0)	1.05 (1.02 - 1.08)	0.001
Age ≥65 years – no./total no. (%)	23/37 (62.2)	190/431 (44.1)	1.57 (0.72 - 3.45)	0.26
Female sex – no./total no. (%)	13/37 (35.1)	142/431 (32.9)	1.03 (0.51 - 2.05)	0.941
Pre-ICU state of health				
Charlson comorbidity index – median (IQR)	4.0 (3.0-5.0)	3.0 (1.0-4.0)	1.28 (1.15 - 1.43)	<0.001
High comorbidity[a] – no./total no. (%)	32/37 (86.5)	309/431 (71.7)	1.84 (0.61 - 5.59)	0.281
Comorbidities				
Hypertension – no./total no. (%)	31/37 (83.8)	240/431 (55.7)	3.90 (1.63 - 9.35)	0.002
Obesity – no./total no. (%)	13/37 (35.1)	159/431 (36.9)	1.02 (0.52 - 2.03)	0.945
Diabetes – no./total no. (%)	14/37 (37.8)	130/431 (30.2)	1.19 (0.60 - 2.37)	0.614
Asthma – no./total no. (%)	1/37 (2.7)	25/431 (5.8)	0.52 (0.07 - 3.80)	0.52
Cancer – no./total no. (%)	5/37 (13.5)	21/431 (4.9)	2.26 (0.85 - 6.00)	0.1
Chronic obstructive pulmonary disease – no./total no. (%)	3/37 (8.1)	17/431 (3.9)	2.46 (0.75 - 8.09)	0.139
Heart failure – no./total no. (%)	6/37 (16.2)	18/431 (4.2)	3.73 (1.52 - 9.12)	0.004
Chronic renal disease – no./total no. (%)	6/37 (16.2)	15/431 (3.5)	4.08 (1.61 - 10.30)	0.003
History of a cerebrovascular accident – no./total no. (%)	2/37 (5.4)	24/431 (5.6)	0.96 (0.23 - 4.02)	0.951
Critical illness				

Risk of death at ICU admission[b], – median (IQR)	32.5 (24.2-39.8)	31.0 (24.0-39.0)	1.00 (0.97 - 1.02)	0.77
Sepsis or septic shock at ICU admission – no./total no. (%)	2/37 (5.4)	9/431 (2.1)	2.39 (0.57 - 9.93)	0.232
Organ dysfunctions during ICU stay				
Delirium – no./total no (%)	9/37 (24.3)	93/431 (21.6)	1.12 (0.53 - 2.38)	0.761
Need of non-invasive mechanical ventilation – no./total no. (%)	9/37 (24.3)	139/431 (32.3)	0.69 (0.31 - 1.53)	0.364
Need of low-flow oxygen therapy – no./total no. (%)	9/37 (24.3)	167/431 (38.7)	0.43 (0.20 - 0.93)	0.43
Need of high-flow oxygen therapy – no./total no. (%)	22/37 (59.5)	304/431 (70.5)	0.55 (0.28 - 1.09)	0.085
Need of invasive mechanical ventilation – no./total no. (%)	16/37 (43.2)	204/431 (47.3)	0.93 (0.47 - 1.83)	0.83
Need of vasopressor – no./total no. (%)	15/37 (40.5)	191/431 (44.3)	0.85 (0.44 - 1.63)	0.623
Need of renal replacement therapy – no./total no. (%)	3/37 (8.1)	25/431 (5.8)	1.37 (0.42 - 4.46)	0.602
Need of blood or blood products transfusion – no./total no. (%)	4/37 (10.8)	54/431 (12.5)	0.83 (0.29 - 2.34)	0.721
Need of parenteral nutrition – no./total no. (%)	1/37 (2.7)	6/431 (1.4)	2.41 (0.31 - 18.76)	0.399
Length of ICU stay, days – median (IQR)	7.0 (2.0-20.0)	8.0 (3.0-17.0)	1.0 (0.97 - 1.02)	0.762
Any-ICU acquired infections[c] – no./total no. (%)	15/37 (40.5)	160/431 (37.1)	0.53 (0.17 - 1.61)	0.264
Pneumonia – no./total no. (%)	15/37 (40.5)	141/431 (32.7)	1.62 (0.66 - 3.98)	0.288
Bloodstream infection – no./total no. (%)	9/37 (24.3)	68/431 (15.8)	2.06 (0.76 - 5.58)	0.154

CI, confidence interval; ICU, intensive care unit; IQR, interquartile range (p25-p75).

[a] Charlson comorbidity index ≥2.

[b] The risk of death was calculated using established prediction equations for hospital death according to the Simplified Acute Physiology Score-2.

[c] Pneumonia, bloodstream infection, or urinary tract infection according to the European Centre for Disease Prevention and Control criteria.
